# Recent Advances in the Preparation of Delivery Systems for the Controlled Release of Scents

**DOI:** 10.3390/ijms24054685

**Published:** 2023-02-28

**Authors:** Adrian Saura-Sanmartin, Laura Andreu-Ardil

**Affiliations:** 1 Departamento de Química Orgánica, Facultad de Química, Universidad de Murcia, 30100 Murcia, Spain; 2 Facultad de Veterinaria, Universidad de Murcia, 30100 Murcia, Spain

**Keywords:** controlled release, profragrances, scents, stimuli-responsive materials, stimuli-responsive molecules

## Abstract

Scents are volatile compounds highly employed in a wide range of manufactured items, such as fine perfumery, household products, and functional foods. One of the main directions of the research in this area aims to enhance the longevity of scents by designing efficient delivery systems to control the release rate of these volatile molecules and also increase their stability. Several approaches to release scents in a controlled manner have been developed in recent years. Thus, different controlled release systems have been prepared, including polymers, metal–organic frameworks and mechanically interlocked systems, among others. This review is focused on the preparation of different scaffolds to accomplish a slow release of scents, by pointing out examples reported in the last five years. In addition to discuss selected examples, a critical perspective on the state of the art of this research field is provided, comparing the different types of scent delivery systems.

## 1. Introduction

Scents are essential components in a wide range of products which are consumed on a daily basis [1]. Thus, consumers can enjoy fragrances in a broad pool of areas, from the pleasant aroma of a fine perfume to the smell of detergents and softeners employed for washing and caring of clothes, but also many other areas, such as the food industry and body care products. This broad representation of aromas in several key industries illustrates the relevance and multivalence of the perfume industry itself [2,3]. Although in the beginning of perfumery only fragrances from natural sources were used, less than 5% of perfume ingredients come from natural sources nowadays [1]. The introduction of synthetic fragrance ingredients marked the starting point of the modern perfume industry, devoting efforts in two main directions: (i) synthesis of the ingredients originally obtained from natural sources, also known as natural identical ingredients; (ii) chemical modification of the natural identical ingredients to obtain a pool of derivatives having an analogous structure but with enhanced properties. The use of both natural and synthetic scents in fine perfumery, flavors, body care, and several household products, among other items, has an inherent handicap due to an intrinsic property of these types of compounds. Scents are characterized for their low molecular weights which allow their efficient evaporation [4,5,6]. Although the volatility is a requirement in order to enjoy the pleasant aroma of these substances, this property could also be a problem, shortening the persistence of the odor. Thus, the useful life of the product can also be reduced during its storage, not only from the starting date of consumption. With the aim of increasing the longevity of the fragrances, numerous scientists have dedicated their efforts in the development of selective and effective release systems, also known as profragrances, which allow the controlled and slow release of extremely volatile scents [7,8,9,10]. Thus, through these systems, the degradation or loss of fragrance is minimized during the process of production and storage, and also the life of use of the product by the consumer is improved.

In this scenario, the development of systems that respond to external stimuli, which has been widely used to build smart materials [11,12,13,14,15] and to control the release of drugs [16,17,18,19,20], among other applications, has turned out to be a suitable strategy in the research focused on scents. These systems have been effectively employed to accomplish a slow release of the fragrance. Different approaches have been developed, including reversible encapsulation, supramolecular interactions, and cleavable covalent bonds [8]. These systems allow the release of scents in response to different stimuli. It is especially important that these stimuli are attributable to conditions which occur spontaneously in everyday use conditions, and also allow the adequate conservation of the organoleptic properties until the consumer starts using the product.

In terms of the adequate applicability, these systems must fulfil a series of requirements: (i) the design of the controlled release system should allow the sustained release according to the specific application; (ii) the release of the scent from the system should be easily controllable without causing damages to the fragrance; (iii) the system should be biocompatible.

This review article aims to provide insights on different systems which can be used for the controlled release of scents by pointing out examples reported in the last five years (2018–2022). The manuscript is divided into seven main sections that analyze different approaches, including materials, small molecules, and supramolecular systems. In a final section, a critical perspective of the state of the art of this research area is provided.

## 2. Polymer- and Gel-Based Release Systems

Natural and synthetic polymers provide high versatility in order to release scents in a controlled manner [21,22,23]. On the one hand, natural polymers usually have high affinity with several scents, beneficiating their stabilization within the polymeric matrix. On the other hand, synthetic polymers lead to a tailorable design, affording different functionalization which is incorporated in the polymeric scaffolds to obtain enhanced release operation.

**Figure 1:**
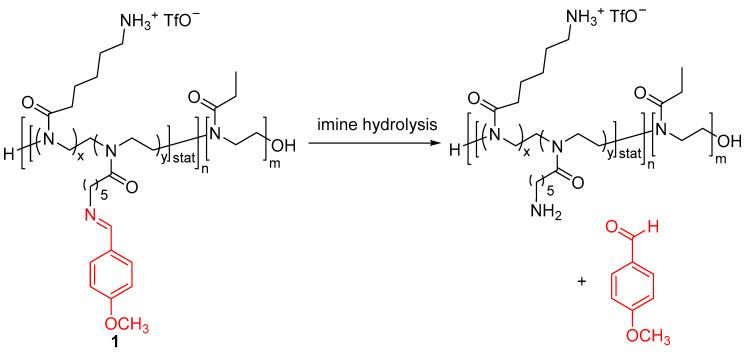
Release of target scent from polymeric profragrances **1** which form micelles in water. The precursor of *p*-anisaldehyde is highlighted. The original results were reported by Jiang and coworkers [27].

The reversible micro- and nanoencapsulation of scents, which has been effectively accomplished by using other materials such as zein nanoparticles, graphene oxide-silica hybrid capsules and chitosan-cellulose particles [24,25,26], has been extensively used by forming polymer–scent conjugates. Indeed, this approach is one of the most common strategies in order to obtain a controlled release of a fragrance. Jiang and coworkers reported pH-responsive pseudopeptide polymeric micelles which effectively deliver fragrances at acidic pH [27]. These systems consisted of copolymers **1** having hydrophilic poly(2-ethyl-2-oxazoline) scaffolds (Figure 1). The Schiff base motif has been grafted as an acid-labile precursor of *p*-anisaldehyde, a fragrance with a very intense smell. This motif also has a different role acting as a hydrophobic pendant group to form the polymer–aldehyde conjugates, providing an amphiphilic nature which led to the self-assembly of micelles in water. These polymeric conjugate-based profragrances were prepared through the grafting of *p*-anisaldehyde by reacting this aldehyde compound with deprotected poly(2-oxazoline) derivatives (POX) bearing amino groups in the presence of triethylamine, leading to the formation of imine bonds. The release mechanism of the aldehyde fragrance operates by a pH input. At acidic pH (pH 5.0), the gradual hydrolysis of the imine linkages placed at the polymers took place, slowly releasing *p*-anisaldehyde over time. This hydrolysis reaction was hindered by hydrophobic interaction provided by the core–shell arrangement, leading to a sustained release time up to 120 h. Remarkably, the polymeric aggregates remain stable during the time and conditions of the release process as confirmed by dynamic light scattering (DLS) and transmission electron microscopy (TEM). This pH responsiveness was tested by studying the release at pH 5 and neutral conditions, observing an enhancement of the scent release in the acidic medium. Interestingly, a high affinity of these profragrances with cotton surfaces was achieved because of the presence of ammonium and tertiary amide groups in the polymeric matrix. Noteworthy, the highest deposition percentage of scent (57.7 ± 8.3%) on the cotton fabrics was achieved using the polymeric conjugate with the highest surface charge.

**Figure 2:**
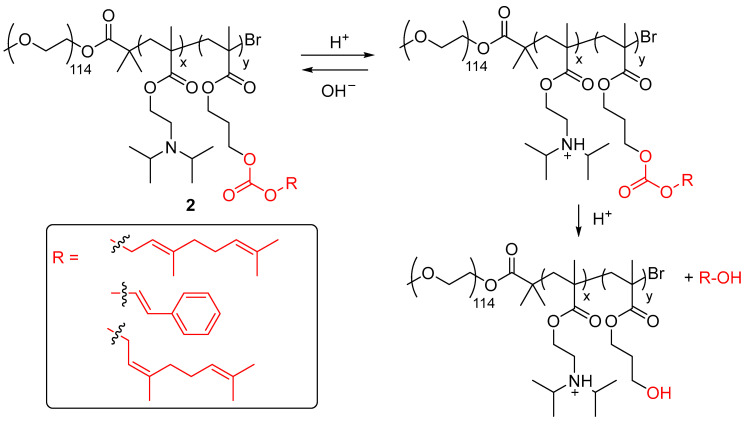
Chemical transformation of the polymeric profragrances **2** by the application of acid/base stimuli. The precursor of alcohol fragrances is highlighted. The different substituents of the alcohol fragrances tested by the authors are included in a box. The original results were reported by Zhu and coworkers [28].

Zhu and colleagues also reported the preparation of polymeric micelles profragrances which operated through a pH stimulus [28]. These systems **2** consisted of hydrophilic polyethylene glycol (PEG) motifs, ionizable hydrophobic pH-activated tertiary amine scaffolds, and pH-triggered carbonic ester units having attached the precursors of the scents (Figure 2). The PEG motif played a dual role in both the stabilization of the polymeric micelles and the physical protection of the inner alcohol scent precursors. Diisopropylamino groups decorated the polymeric matrix in order to provide switchability to the systems, but also offered a hydrophobic core to the inner profragrance. In basic medium, the polymer-scent conjugates formed micelles, encapsulating the profragrances. The reduction of pH led to the dissociation of the polymeric micelles by the protonation of the tertiary amine motifs, exposing the attached scents to the medium. Interestingly, by increasing the pH of the medium, the micelle state can be recovered. The “off” state of the polymeric micelles was determined at pH > 6.4. Thus, a protective effect of the micellar architecture avoided the hydrolysis of the carbonic ester group at pH 10 and pH 6.6, in which this functionality is unstable, resulting in no alcohol scent release upon these conditions. Further increase in the acidity of the medium led to the release of the alcohol derivatives-based scents attached in the carbonic ester units. The release of these scents was measured over time by using gas chromatography with a flame ionization detector (GC-FID). Interestingly, the aroma was also perceivable through the sense of smell. The PEG scaffold was also used by Xiao, Zhang and coworkers in the preparation of biocompatible nanocapsules which prolong the release time of lily fragrance [29].

**Figure 3:**
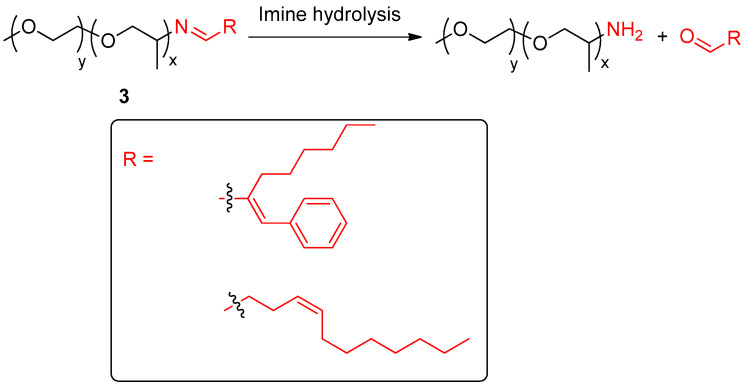
Release of aldehyde-based scents from profragrances **3**, highlighting the aldehyde scent precursor unit. The fragments of the scents are included in a box. The original results were reported by Giuseppone, Herrmann and coworkers [30].

Giuseppone, Herrmann and colleagues reported the polymeric cleavable surfactants imines **3** (Figure 3) which effectively released different aldehyde-based scents [30]. These amphiphilic imine profragrances operated as surfactants solubilizing target scents in aqueous medium by forming micelles. Noteworthy, the imine functionality turned out to be notably stable in surfactant solutions in water. This high storage stability in aqueous medium is advantageous to exploit potential applications in functional perfumery. The release of the aldehyde scents was studied by using dynamic headspace measurements, showing a fragrance evaporation similar to that of the PEG25 monostearate which was employed as model. Interestingly, the evaporation of water and perfume induced a rapid cleavage of the surfactant, leading to a blooming effect which increments the intensity of the fragrance. The preparation of binary mixture of cleavable and non-cleavable surfactants led to an increased boosting of scent perception compared to that of the single cleavable surfactant. The authors went a step further and prepared a model shower gel formulation containing their profragrance-based systems and two additional surfactants, the anionic sodium pareth sulphate and the amphoteric cocamido betaine. Dilution of this concentrated formulation led to an effective and fast hydrolysis of the imine, releasing the aldehyde-based scents. Interestingly, this dilution promoted a more balanced overall fragrance evaporation than that observed in the non-diluted pristine solution. Indeed, the imine bond was stable in the non-diluted shower gel formulations at room temperature for 39 days. This high stability towards the storage of the profragrance in aqueous formulations paves the way to enhanced implementations in both fine perfumery and body care products.

**Figure 4:**
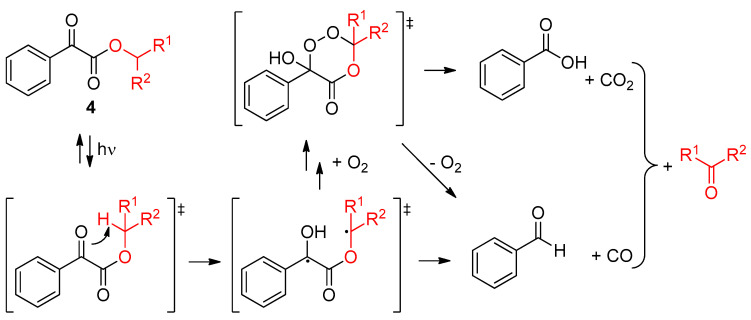
Mechanism of photorelease in profragrances **4** through a Norrish type II photofragmentation. The precursor of the aldehyde- or ketone-based scent is highlighted. The original results were reported by Herrmann and coworkers [31].

Herrmann and coworkers also reported the microencapsulation of scents using the photoresponsive 2-oxoacetates-based profragrances **4** [31]. The profragrances were composed of a 2-oxo-2-phenylacetate motif having attached the precursor of the scent. Upon light exposure, a Norrish type II photofragmentation took place, releasing volatile aldehyde or ketone-based scents (Figure 4). An interfacial polymerization of a melamine-formaldehyde resin in water phase with polyisocyanate and the corresponding profragrance **4** afforded the core-shell microcapsules containing the scents precursors. The polymeric encapsulation of the profragrances led to a reduction in the release rate of scent in comparison to that of the non-encapsulated profragrances **4**. This photorelease mechanism can proceed at moderate light intensities, resulting in its applicability in everyday use. Since profragrances are usually formulated together with perfumes, which contain different compounds having a wide range of functional groups, the authors also studied different combinations of profragrances **4** with model perfumes, observing that the encapsulation of compounds which affect the efficiency of the photoreaction led to a deceleration of the release of the target scents. Thus, compounds interfering the photo-triggered release kinetics should be avoided if a fast light-induced release is pretended and should be added when a slow release is desired. The same research group also reported the application of photoresponsive polystyrene-based 2-oxoacetates for the controlled release of different scents deposited onto a cotton surface in a fabric softening process [32] and multi stimuli-responsive core/shell microcapsules containing 2-oxoacetates which release volatile compounds in a controlled manner [33].

Choe, Roh and colleagues prepared thermoresponsive semi-interpenetrating gelatin-alginate networks for encapsulation and controlled release of different meat-like flavors [34]. The systems were prepared by addition to scents to a homogenized mixture of gelatin and alginate previously incubated in water. Alginate molecules reinforced the gelatin-based hydrogel by establishing a semi-interpenetration that provided a higher resistance to external stimuli. Thus, a sustained release of the meat scents was accomplished at a define temperature. Noteworthy, the scents remained intact during the refrigerated storage, paving the way to enhanced applications in food industry.

Also using encapsulation methods, zwitterionic comb-like lipid polymers have turned out to be suitable scaffolds in order to prolong the fragrance retention [35]. Indeed, the encapsulation efficiency of linalool was enhanced by using these polymers in comparison to that of PEG-based ones. Thus, after 24 h, 24.49% of linalool was released from the PEG-based capsules and a value of 16.83% was detected in the case of the zwitterionic lipid polymeric capsules.

Low molecular weight gels, which are obtained from a matrix of self-assembled small molecules by the establishment of non-covalent interactions, construct anisotropic structures able of immobilizing different molecules within the supramolecular network [36,37,38,39,40], thus being useful in order to accomplish sustained release of scents.

**Figure 5:**
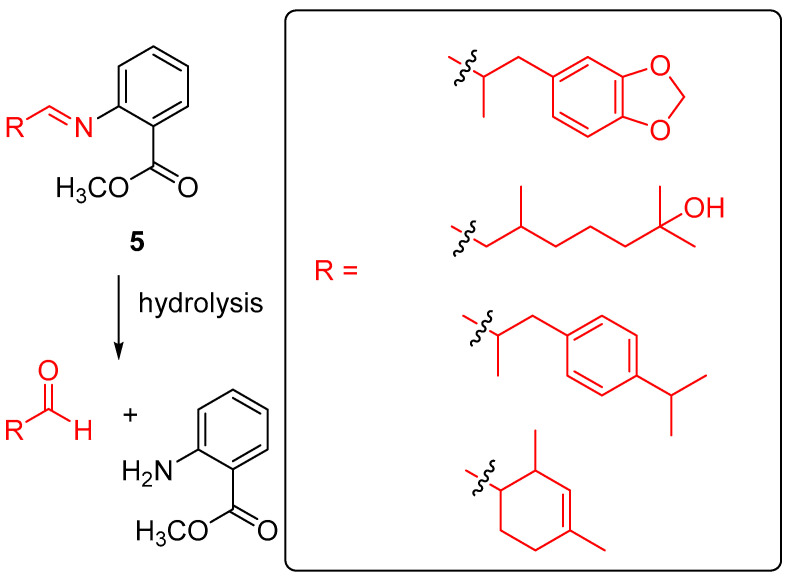
Release of aldehyde-based scents from profragrances **5**, highlighting the aldehyde scent precursor unit. The fragments of the scents are included in a box. The original results were reported by Tomasini, Giuri and coworkers [41].

Tomasini, Giuri and coworkers carried out the uptake of the Schiff bases profragrances **5** (Figure 5) in low molecular weight gels, prepared by L-Dopa-based gelator derivatives functionalized with ester or carboxylic motifs, obtaining a high loading of scents [41]. The profragrances were prepared through the reaction of methyl anthranilate and the target racemic mixtures of the aldehydes by direct condensation of these reactants at 110 °C. The instability of the imine bond impeded the purification of most of the systems, except for one of them which was isolated upon crystallization using methanol. However, due to the high conversion towards the Schiff bases profragrances, the unpurified substances were employed in the release study. By decreasing the pH of the medium, control over the release rate of these profragrances was accomplished by changing the hydrolysis degree of the imine functionality, which delivered the aldehyde-based scents in ethanol/water mixtures. Interestingly, the preparation of low molecular weight gels through the solvent switch method, by mixing compounds **5** and two L-Dopa-based gelators in ethanol and then adding water to accomplish gel formation, led to a higher control of the hydrolysis rate of the imine bonds. Thus, the profragrance-loaded gels afforded a slower release in comparison to that of the free Schiff bases. Noteworthy, the employment of the gelator-having ester groups showed a really slow scent release perduring weeks but with smaller intensities, while the gelator-bearing acidic motifs provided a faster fragrance release compared to the other gelator, being useful in applications which require the scent release to take place in a few days. These results pave the way for the development of fine-tunable scent supramolecular delivery systems.

Supramolecular chemistry plays a key role in the formation of some polymers and gels, as it is the case of the anisotropic networks obtained in low molecular weight gels, but it can also play a direct role by the establishment of non-covalent bonds with the scent. Among the non-covalent interactions, hydrogen bonding has been postulated as a very useful tool in supramolecular chemistry. Hydrogen bonds are characterized by their directionality and their great ability to act cooperatively, forming different associations. When designing controlled release systems which operate through the establishment of hydrogen bonds, control over the dissociation of the different components is feasible using solvent changes as stimuli or other compounds that interact more strongly than the original supramolecular association [42,43,44,45,46].

**Figure 6:**
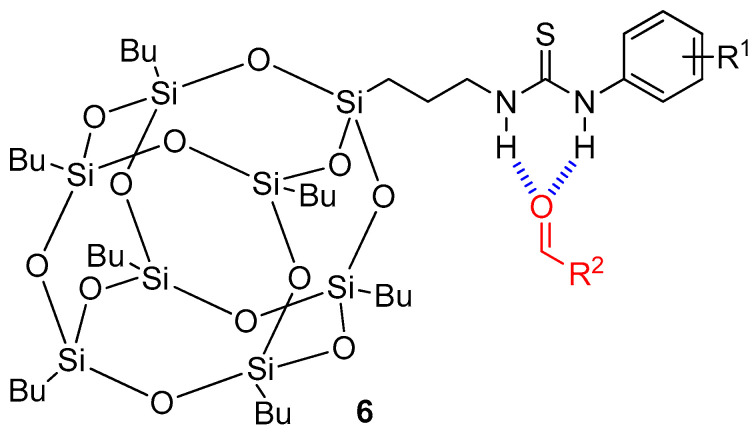
Chemical structure of thiourea-based profragrances **6**, highlighting the aldehyde scent and the hydrogen bonds. The original results were reported by Zhu and coworkers [47].

Zhu and colleagues employed the thiourea-functionalized polyhedral oligomeric silsesquioxane (POSS) **6** as a supramolecular profragrance system (Figure 6), forming hydrogen bonds with different aldehyde-based scents [47]. The synthesis of the oligomeric-based thiourea was carried out by reaction of aminopropylisobutyl POSS and the corresponding aromatic isocyanate using dichloromethane as solvent, affording the target products in high yields (85–98%). POSS precursors offer different advantages in order to achieve a direct application, such as nontoxicity and great affinity to wallpaper fabrics. The spontaneous formation of bifurcated hydrogen bonds between aldehyde scents and thiourea-based POSS was monitored by ^1^H NMR experiments of aldehyde-based scents:POSS solutions in a 1:1 ratio using dry CDCl_3_ as solvent, observing a shift of the hydrogen of the aldehyde functional group of the scents, thus indicating the successful formation of the supramolecular interaction. The ambient humidity is sufficient for the water molecules existing in the air to establish stronger hydrogen bonds with the thiourea unit, thus slowly releasing the target aroma. Additionally, electron-withdrawing groups, such as trifluoromethyl and nitro groups, placed at the aromatic ring of the thiourea-functionalized POSS enhanced the scent release in comparison with electron-donating groups. Interestingly, the authors embedded the profragrances in wallpapers, demonstrating the desired controlled release of the supramolecular oligomeric profragrance in comparison to that embedded with pure aldehydes.

## 3. Ionic Liquid-Based Release Systems

Ionic liquids are solvents consisting of bulky and unsymmetrically substituted organic cations and anions which are in the liquid phase at temperatures below 100 °C [48]. Ionic liquids have been employed extensively in biomedical applications and as electrolytes, but also in controlled release systems and as excipients to solubilize a wide range of small molecules in water [49,50,51,52,53,54,55,56,57]. Ionic liquids interact with the solute and disrupt the interactions of the solute with a co-solvent, integrating the molecule into a space provided by this type of molecular system. Regarding fragrance release applications, ionic liquids usually have negligible vapor pressure at ambient conditions which would potentially render excellent profragrance scaffolds.

**Figure 7:**
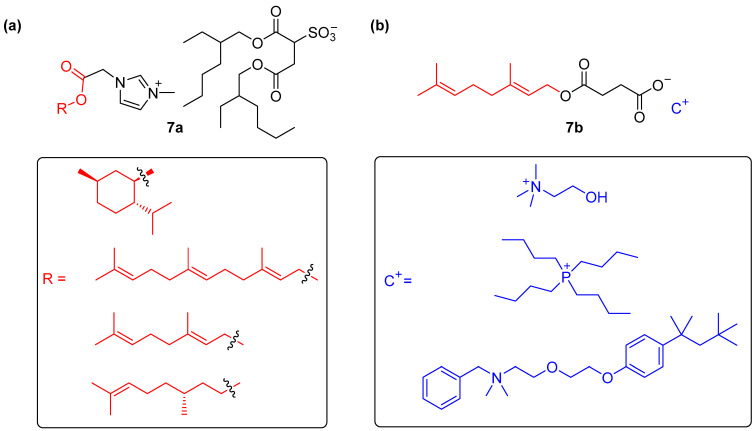
(**a**) Chemical structure of the ionic liquid profragrances **7a**, highlighting the precursor of the alcohol scent and showing the fragments of the scents included in a box; (**b**) chemical structure of the ionic liquid profragrances **7b** including the different cations in a box. The original results were reported by Rogers and coworkers [58]. The ester hydrolysis reaction which provides the target alcohol-based scents has not been included in the figure.

Rogers and coworkers developed the ionic liquid-based profragrances **7a** constituted by 3-ethyl-1-(alkyloxycarbonylmethyl)imidazolium derivatives bearing the scent precursor grafted in a labile ester group and sodium docusate (Figure 7a) [58]. These ionic liquid-based profragrances were prepared in quantitative yields showing high purities (>90%) by first forming the imidazolium derivatives followed by a final metathesis reaction with sodium docusate at room temperature. The thermal properties of the profragrances 7a were studied by using thermogravimetric analysis (TGA), observing a two-step decomposition pattern, which is characteristically detected for thermolabile profragrances, when systems incorporation allylic alcohols precursors (geraniol and farnesol) were heated in the presence of moisture, while a single-step decomposition pattern was obtained in the case of the saturated fragrance (citronellol and menthol). These results indicated that allyl ester bonds are required if a thermal hydrolysis of the profragrances is expected to provide the release of the target scents. However, if biocompatible applications are desired, the imidazolium salts turn out to be toxic. In order to overcome this main disadvantage, the authors envisioned the utilization of the liquid ionic **7b** containing a biocompatible geraniol functionalized hemisuccinate motif and different nontoxic counter-cations (Figure 7b). The system having choline hydroxide as cation was dissolved in buffered solutions at pH 3 and 9 at 25 °C and monitored over time by using ^1^H NMR measurements. These experiments revealed a slow release at acidic conditions while a faster hydrolysis rate was obtained at basic conditions, with a complete release of the alcohol scent after 250 min. Interestingly, this strategy towards the synthesis of ionic liquid-based profragrances allows the preparation of a dual system having both cation and anion as active molecules, paving the way to enhanced applications with two different molecules being released.

## 4. Metal-Organic Framework-Based Release Systems

Metal–organic frameworks (MOFs) are a type of porous material which have several advantages over conventional microporous materials, such as the great versatility of their design and being able to easily control the pore size. MOFs are constituted of organic ligands coordinated to metal ions, forming a uniform crystalline lattice, which can be repeated in any of the three dimensions. The chemistry of MOFs has experienced great progress in the last decades due to the possibility of generating almost infinite structures from numerous commercially available metal precursors and an immense range of organic ligands, both commercial and synthesized in the laboratory. Thus, an exponential increase in the research interest in these systems has arisen. The design of the morphology of the crystalline matrix, as well as the fine-tuning of the pore size, has resulted in several applications, such as gas adsorption, heterogeneous catalysis, and selective recognition of a wide range of target molecules [59,60,61,62,63,64,65,66,67,68].

The versatility of these metal–organic porous materials has led to the design of systems that selectively incorporate target molecules within the pores of the crystals and allow the controlled release of the cargo upon the application of different stimuli, such as light, pH, or redox. The switchable delivery is favored by incorporating stimuli-responsive organic struts within the crystalline arrays. Thus, the release of a wide variety of compounds has been attempted by using MOFs as adjustable scaffolds [69,70,71,72,73,74,75,76,77,78,79].

Although the use of other microporous materials operating as scent nanodispenser-based systems such as zeolites has been reported [80,81], MOFs have turned out to be fine-tunable microporous vehicles which allow an enhanced and tailorable control of the release rate of scents.

**Figure 8:**
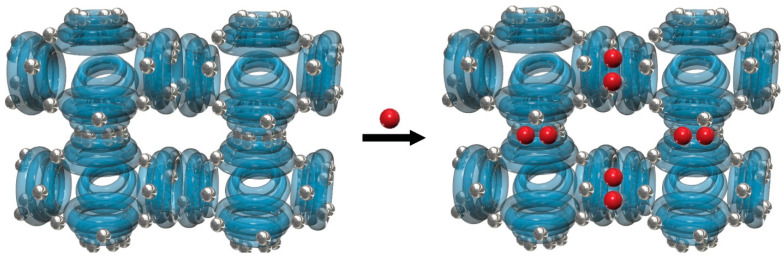
Cartoon representation of the encapsulation of fragrance within the pores of **γ-CD-MOF**. Big spheres represent the target scent, small spheres represent potassium ions, and semitransparent toroid-truncated cones represent γ-CD organic struts. The original results were reported by Zhou and coworkers [82].

Zhou and colleagues reported the encapsulation of volatile scents within the pores of γ-cyclodextrin (γ-CD)-based MOFs [82]. **γ-CD-MOF** nanocrystals were prepared by using γ-CD as organic ligand and potassium hydroxide as a metal source according to a previously reported protocol [83]. In this metal–organic crystalline porous material, six γ-CD are assembled into a six-faced cubic lattice which form a large hydrophilic and spherical pore having 1.7 nm of diameter. Additionally, these nanocrystals also have smaller pores, including hydrophobic pores having a diameter of 1.0 nm, as well as other minor pores. The coordination of potassium ions with hydroxyl groups of the macrocyclic organic struts extends the crystal lattice in all three dimensions. Noteworthy, both γ-CD and **γ-CD-MOF** are biocompatible according to the (3-(4,5-dimethylthiazolyl-2)-2,5-diphenyltetrazolium)bromide cell proliferation test. The fragrance encapsulation of the organic ligands and the metal–organic material was accomplished by soaking both systems in fragrance oil for 12 h (Figure 8). ^1^H NMR experiments allowed the calculation of the loading of ester- and terpenoid-based fragrances, determining a higher loading capacity of the MOF scaffold compared to that of free macrocyclic ligand. The long-term controlled fragrance release was studied by measuring the accumulative release of the target scents over 2 weeks, showing a more retarded release of scents in **γ-CD-MOF**. Interestingly, a dependence on the encapsulated fragrance in the release rate was also observed. The biocompatibility of these systems led to envision potential applications as promising fragrance carriers for different industries, including cosmetics and the food industry.

**Figure 9:**
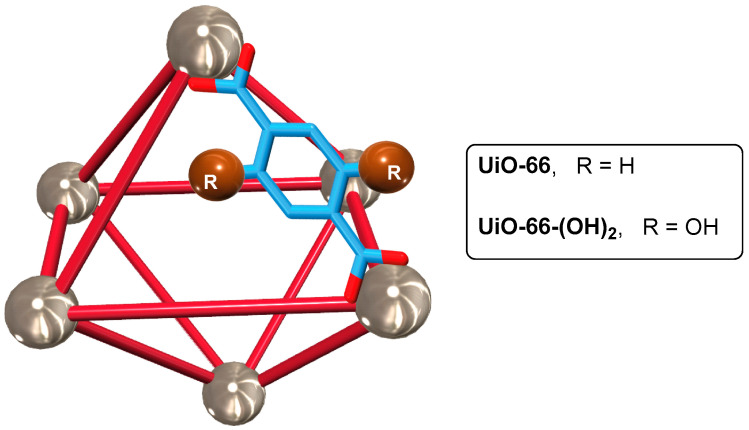
Cartoon representation of the **UiO-66**-based systems employed as scent nanocarriers. Big spheres represent the zirconium clusters, small spheres represent the substituents of the terephthalate derivatives, and rods represent the terephthalate ligands. For clarity, one of the ligands is showed as a modelized chemical structure. The original results were reported by Zhou and coworkers [84].

The same research group also reported the encapsulation and controlled release of ester- and terpenoid-based scents using two different **UiO-66**-based zirconium organic frameworks [84], prepared following previously reported synthetic protocols [85,86]. Both metal–organic nanocarriers, **UiO-66** and **UiO-66-(OH)_2_**, contain two types of voids in their structure, eight small tetrahedral voids surrounding a large octahedral one, formed by the coordination of terephthalic acid derivatives and zirconium clusters (Figure 9). The effect of the surface functionalization of MOFs on scent encapsulation was evaluated by using polar esters and nonpolar terpenoids as fragrance models, observing that the loading capacity is affected by the chemical structure, molecular size, and vapor pressure. **UiO-66-(OH)_2_** afforded a higher loading capacity of polar fragrances as a result of the strong interactions between polar molecules and the polar metal–organic nanocarrier. Thus, ester groups could form hydrogen bonds with the hydroxyl groups of the organic struts of the functionalized MOF, enhancing the uptake of these polar fragrances. These strong hydrogen bonds also regulated the release of the ester scents, leading to half-release times of the selected scents in the range of 2.5–15 days at room temperature. These MOF-based nanodispensers exhibited enhanced sustained release properties, suggesting that the hydroxyl groups in the MOFs improved the encapsulation capacity and lengthened the release time because of the establishment of noncovalent interactions.

Li, Wang and coworkers also employed **UiO-66** as nanocontainer of three different food-flavoring agents, isophorone, eugenol, and β-ionone [87]. Following a previously reported protocol [88], the zirconium–organic framework was prepared, having a pore size of 6.7 nm and a pore volume of 0.12 cm^3^/g, determined by N_2_ adsorption-desorption experiments. The encapsulation of the scents was accomplished by dispersing **UiO-66** nanocrystals in ethanol and cyclohexane and subsequently adding the target scent. The sustained release was evaluated by measuring the loading amount of cargo after 5 h at different temperatures (15–90 °C). A remarkable decrease of the loading amount was obtained with the increment of the temperature. A long-time release study was also carried out at room temperature, observing a sustained release during the time of the experiment (20 days). Thus, control over the release rate could be obtained by simply changing the temperature, adjusting the system to different applications.

Biocompatible **MIL-101-NH_2_** has been post-synthetically functionalized to afford an effective tuning of the release rate of volatile compounds [89]. Different host–guest interaction affinities were afforded as a result of the functionalization, leading to distinct sustained release profiles. Thus, the release rate of scents can be prolonged for days to months and, noteworthy, over a year (half release times from 1.1 to 674.8 days). The **MIL-101**-based MOFs have been also employed in the development of high-performance nanoporous carbon which displays selective adsorption-release of different fragrances [90].

## 5. Organic Salt-Based Release Systems

The water-triggered release systems offer the advantage that the moisture of the air can act as a stimulus in order to achieve a slow release of the target scents. Thus, the hydrolysis of profragrances is a suitable strategy to design sustained release systems. In this scenario, hydrolyzable organic salts, which incorporate the scent precursor in their structure, have become suitable candidates to be used as profragrances.

**Figure 10:**
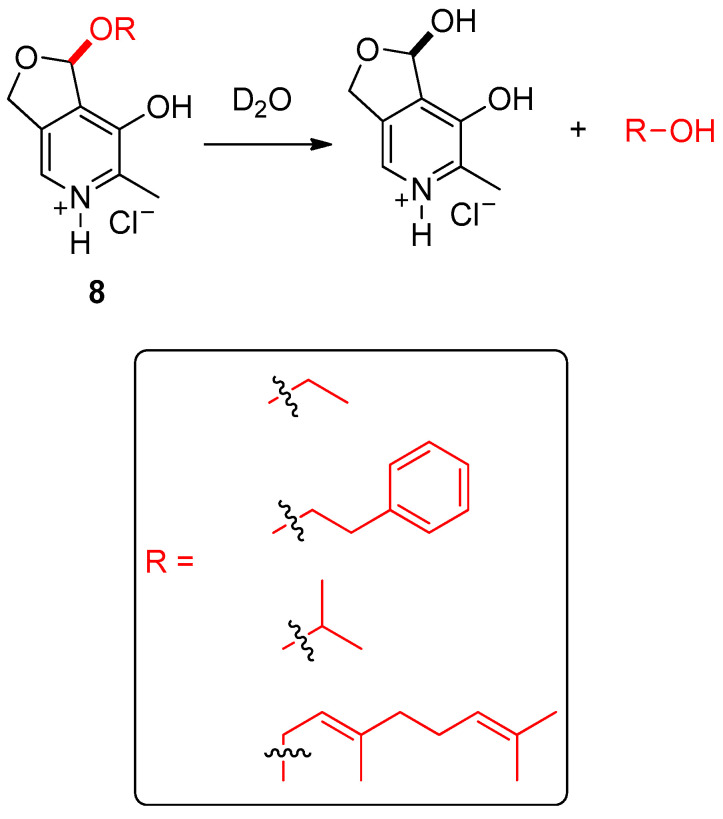
Water-triggered release of alcohol-based scents from pyridoxal acetal profragrances **8**, highlighting the precursor of the alcohol scent and showing the fragments of the scents include in a box. The original results were reported by Boyce and coworkers [91].

Boyce and colleagues reported the employment of the pyridoxal acetal salts **8** as vitamin-based profragrances which effectively released alcohol-based scents in the presence of water at neutral pH (Figure 10) [91]. These systems were prepared from pyridoxal·HCl through the thermal treatment (60 °C) in the presence of the target alcohol. The increment in the temperature first formed o-pyridinone methide which then reacted via oxa-Michael addition with the corresponding alcohol, providing the pyridoxal acetal scaffold. Interestingly, this reaction proceeded under mild conditions due to the stabilization provided by the dihydrofuran motif, leading to the formation of the profragrances in high yields in the absence of catalyst. Noteworthy, due to the clean conversion, the purification step was avoided. The isolation of the pure products was thus achieved by simple evaporation of the solvent in the case of the volatile alcohols (ethanol and isopropanol) and by precipitation using diethyl ether in the case of the higher boiling point alcohol-based scents (geraniol and 2-phenylethanol). The rate of the scent release was monitored by time-dependent ^1^H-NMR studies at 0.2 M concentration in a mixture of D_2_O and DMSO-*d_6_*. The hydrolysis of the salts **8** quantitatively afforded the target alcohol fragrance and pyridoxal·HCl, observing the highest release (37%) after 22 h in the case of ethanol measured in 30% of D_2_O in DMSO-*d_6_*. Remarkably, the release rate can be modulated by changing the proportion of D_2_O in the mixture of solvents. Thus, higher concentrations of D_2_O led to faster release rates than those obtained using lower concentrations.

Surfactants are a type of organic compounds having, in the same molecule, both hydrophobic and hydrophilic scaffolds, providing an amphiphilic nature which can form supramolecular associations. This inherent characteristic has led to several applications, such as detergency, food processing, and cosmetics formulation [92,93,94,95]. Surfactants also have potential applications in the perfume industry, increasing the aroma perception time by providing a decrease in the volatility of the aroma.

**Figure 11:**
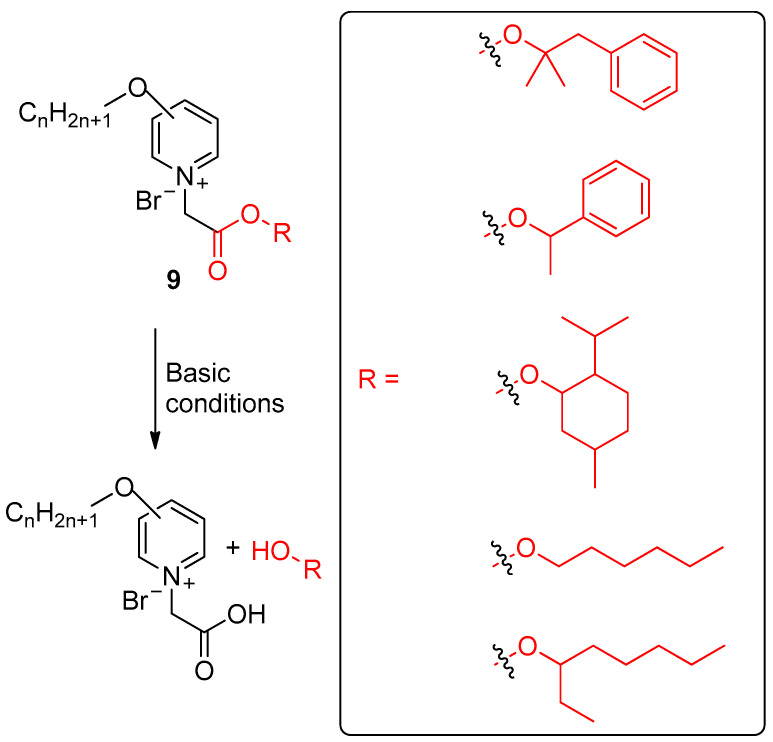
Release of alcohol-based scents from quaternary pyridinium surfactants profragrances **9**, highlighting the precursor of the scent. The fragments of the scents are included in a box. The original results were reported by Han, Zhu and coworkers [96].

Han, Zhu and colleagues developed the profragrances **9** in which the alcohol-based scents are linked with quaternary pyridinium salts (QPS) by an ester group (Figure 11) [96]. These salts are biodegradable cationic surfactants which can act as germicides by perturbing the bacterial lipid bilayer membranes on Gram-positive bacteria. The synthesis of the QPS-based profragrance systems started with the reaction of 3-hydroxypyridine or 4-hydroxypyridine with brominated alkanes in the presence of KOH using DMSO as solvent, leading to 3-alkoxypyridines and 4-alkoxypyridines. Subsequently, the second synthons were prepared by the reaction between bromoacetyl bromide and the target alcohol derivatives. This esterification reaction was carried out in the presence of pyridine using dichloromethane as solvent. Finally, a quaternization reaction afforded the target QPS profragrances from the alkoxypyridine derivatives and the previously synthesized esters using toluene as solvent. By this way, it is possible to synthesize 24 QPS profragrances with different chemical structures. The fragrance alcohols employed to accomplish the controlled release were methylbenzyl alcohol, 1-phenylethanol, L-menthol, 1-hexanol, and 3-octanol. The QPS profragrances allowed the release of these alcohols under alkaline conditions over a long time. The analysis of the surface activity of QPS showed a surface tension range from 20.6−34.9 mN m^−1^ and 19.5−33.7 mN m^−1^ for 3-substituted pyridinium derivatives and 4-substituted pyridinium derivatives, respectively. The critical micelle concentration (CMC) values of these QPS profragrances were between 0.003 and 0.492 mmol L^−1^. The calculation of the CMC/C20 value, which indicates the adsorption and micellization capacities at the interface, showed a very low value indicating the formation of micelles by decreasing the surface tension of water in the case of 3-substituted QPS, while 4-substituted QPS had a higher value, which indicated that these systems favored the adsorption instead of the micellization. The authors carried out a controlled release study using the moisture of a humid environment as external stimulus to promote the release of the scents and headspace solid phase microextraction and gras chromatography as analytical techniques. Noteworthy, different releasing rates were obtained as a consequence of the different steric hindrance provided by the substituents closed to the ester bond, which can hinder the hydrolysis reaction. Interestingly, a slow release was obtained in the case of the 4-substituted derivatives, as a consequence of the electronic effect of the *p*-alkoxy motif over the pyridine ring.

## 6. Coumarin-Based Release Systems

Coumarin derivatives are organic chemical compounds of the benzopyrazone family which have been widely employed as photoactive systems for the delivery of drugs in a controlled manner [97,98,99,100,101]. This photo-triggered release of prodrugs has led to envision the use of coumarin-based compounds as profragrances.

**Figure 12:**
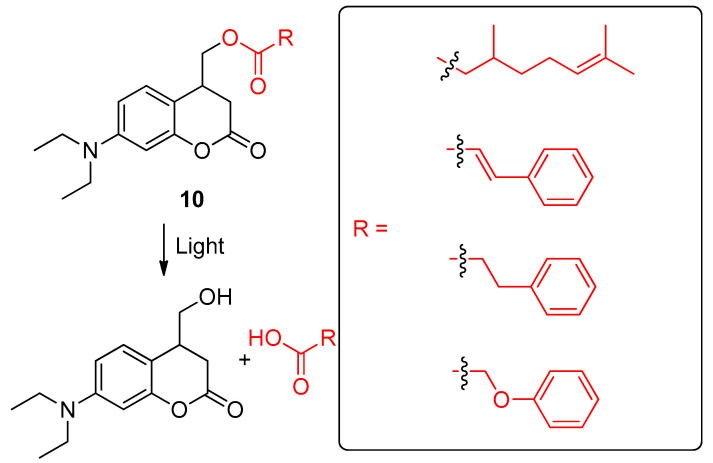
Photo-triggered release of carboxylic acid-based scents from coumarin-based profragrances **10**, highlighting the precursor of the scent. The fragments of the scents are included in a box. The original results were reported by Han, Zhu and coworkers [102].

Han, Zhu and colleagues designed a new family of photo-triggered 4-hydroxymethyl coumarin derivatives-based profragrances (**10**) in which volatile carboxylic fragrances were grafted in the scaffold forming an ester bond [102]. These systems allowed the slow release of the scent derivatives through the photo-triggered cleavage of the ester bond upon light irradiation (Figure 12). This light-activated rupture of the ester bond which effectively released the target scents was confirmed using NMR titration, HPLC, and MS titration experiments. The coumarin scaffold allowed the release of the fragrance molecules under the application of low intensity visible light, such as that of sunlight. Moreover, the unprecedented fluorescence intensity of the coumarin derivative which is generated after the release of the scent can be employed in order to monitor the release of carboxylic acid-based fragrance molecules. The synthesis of the profragrances **10** was accomplished by the selective oxidation of 7-diethylamino-4-methylcoumarin with selenium dioxide, followed by a reduction reaction using sodium borohydride and a final esterification in a dark environment with the corresponding carboxylic acid-based fragrances (citronellic acid, cinnamic acid, hydrocinnamic acid, and phenoxyacetic acid) using 1-(3-(dimethylamino)propyl)-3-ethylcarbodiimide (EDC) as condensation reagent and 4-dimethylaminopyridine (DMAP) as base. As abovementioned, the use of coumarin as a fluorophore allows monitoring of its fluorescence signals along the photolysis process. Thus, the research group studied the absorption and emission spectra of the profragrances **10** upon successive irradiation using a Hg/Xe lamp. As results, the release of the systems containing citronellic acid and phenoxyacetic acid showed quicker rates than those of cinnamic acid and hydrocinnamic acid under the same irradiation intensity. The different profragrance systems showed an increased fluorescence upon successive photoirradiation. A linear correlation between fluorescence intensity and the fragrance release rate was determined according to the Korsmeyer-Peppas kinetic model (*R^2^* > 0.95). Noteworthy, the profragrances were thermostable in the solid state under dark conditions. However, the target fragrance molecules were released upon irradiation in solid state and then volatilized, leading to a kinetic curve which indicated that the evaporation rate of profragrance-based coumarins **10** was slower than that of the free carboxylic acid scents. Interestingly, this light-controlled release performance of profragrances was also tested on wallpaper as a model of a practical surface in the case of the citronellic acid derivative, affording similar results to those obtained in the solid state. The same research group also reported the photo-triggered delivery of scents by using coumarin-based profragrances encapsulated within amphiphilic pluronic nanoparticles [103].

## 7. Cyclodextrin-Based Release Systems

Cyclodextrins are a type of truncated cone-like macrocyclic compounds constituted by oligosaccharides connected through α-1,4-glucosidic bonds. CDs have been widely employed in host–guest chemistry, encapsulating target molecules within their cavities. Thus, a rational design of different systems containing CDs, such as interlocked molecules, cross-linked hydrogels, and supramolecular polymers, have led to enhanced applications in the research field of supramolecular chemistry. The possibility of use of CD complexes as controlled release systems has allowed the application of these supramolecular systems in several biocompatible applications, such as functional foods and drug delivery [104,105,106,107,108,109,110]. Thus, in addition to the previously mentioned γ-CD-MOF [82], cyclodextrin macrocycles have been directly employed in the sustained release of scents. Indeed, cyclodextrins have been extensively employed in the preparation of functional food [111], paving the way for their application in the encapsulation and release of flavor and fragrances so that the consumer perceives the unaltered organoleptic properties of the product.

**Figure 13:**
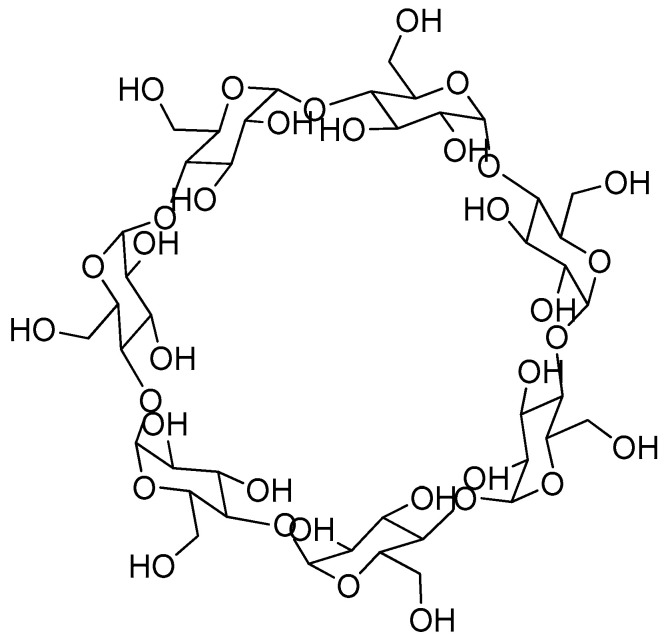
Chemical structure of β-CD.

Niu and colleagues envisioned the encapsulation of apple fragrance within the cavity of β-CD (Figure 13) [112]. The inclusion complexes were prepared by coprecipitation, adding apple fragrance to a solution of β-CD with a 1:8 weight ratio (scent:β-CD). The mixture was cooled at 4 °C overnight and the precipitated powder was subsequently washed and dried. A 5.8% of loading was achieved according to the data measured by TGA. The apple fragrance@β-CD inclusion complexes were impregnated in cotton fabrics which were previously dipped into an aqueous emulsion containing citric acid monohydrate, urea, and sodium bicarbonate. The same content of apple fragrance was also impregnated to cotton fabrics as control model. The slow release of the apple scent of the perfumed cotton fabrics was analyzed over time at room temperature by using gas chromatography-mass spectrometry (GC-MS). A slower release of the apple scent was determined in the case of the inclusion complexes compared to the nonencapsulated fragrance. Noteworthy, enhanced sustained release properties of the cotton fabrics impregnated with apple fragrance@β-CD inclusion complexes were observed through an electronic nose analysis with an effective scent life of 80 days, which is twice as long as that obtained in the nonencapsulated fragrance. Thus, the encapsulation of the scent turned out to be a suitable strategy which protected the scent enhancing its longevity.

The application of inclusion complexes based on β-CD macrocycles predominates in the examples reported in the literature [113,114,115,116,117,118,119]. However, other cyclodextrin derivatives have also been employed as nanocontainers of fragrances. As an example, watermelon flavor@γ-CD inclusion complexes were reported by Kou and colleagues, showing excellent sustained release properties [120]. The inclusion complexes were synthesized by using a freeze–drying method of a mixture of γ-CD:watermelon flavor in a 3:1 ratio. The authors studied the sustained release of the seven main aroma compounds of the watermelon flavor, observing a decrease of inclusion efficacy as a consequence of the different functional groups of the scents. Thus, alcohol-based scents showed the higher encapsulation efficiency in comparison to that of aldehydes, and esters fragrances revealed the lowest inclusion efficiency. The release rate turned out to be dependent of the interaction forces between fragrance components of the flavor and the cavity of cyclodextrin-based macrocycles, thus leading to a reduction of the release ratio with an increment in hydrophobicity.

## 8. Rotaxane-Based Release Systems

Rotaxanes are a type of mechanically interlocked molecules (MIMs) constituted by at least two components: a cyclic counterpart, also known as macrocycle or wheel, surrounding a linear counterpart, also known as axle or thread. These components are not covalently bonded, but rather are stabilized by noncovalent forces, forming a mechanical bond. The dissociation of the different counterparts is prevented by bulky groups at both ends of the thread, also known as stoppers [121,122,123]. The research focused on rotaxanes has attracted the interest of many scientists in the area of supramolecular chemistry due to the new properties conferred by the mechanical bond and also for the dynamics of the counterparts and the possibility of exercising control over this motion. Thus, the chemistry of rotaxanes has resulted in several applications in the area of molecular machinery [124,125,126,127,128,129,130,131,132,133]. A rational design of the rotaxanated architectures has led to the development of biological applications, highlighting the switchable release of anticancer drugs [134,135,136]. The versatility of the structural design of these intertwined compounds has also led to envision their use as effective profragrances.

**Figure 14:**
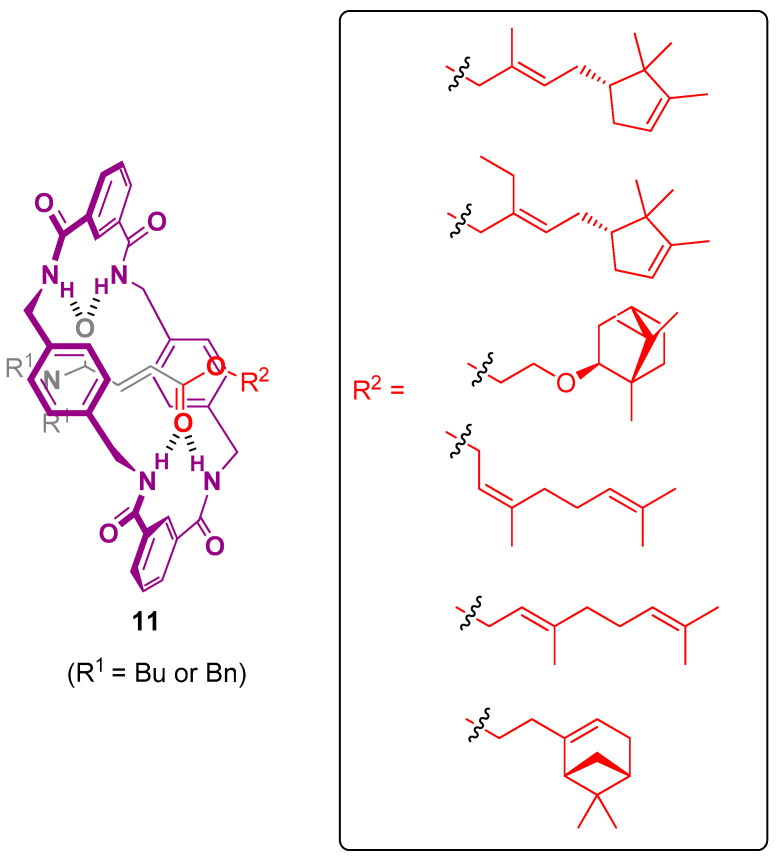
Chemical structure of the interlocked fumaramate-based profragrances **11**, highlighting the scent-based stopper. The fragments of the scents are included in a box. The original results were reported by Pastor, Berna and coworkers [137]. The ester hydrolysis reaction which provides the target alcohol-based scents has not been included in the figure.

Pastor, Berna and colleagues designed the [2]pseudorotaxane **11** scaffolds having a fumaramate bearing a scent-based stopper as a thread and a benzylic amide macrocycle (Figure 14) [137]. The esterification of volatile alcohol-based fragrances and further mechanically interlocking encapsulation within the tetralactam wheel enhanced the stability of the scent providing a lower volatility. Thus, rotaxanes profragrances can provide ideal scaffolds to storage scents retaining the organoleptic properties. The release of scents was conducted by sequential thermal or photochemical dethreading followed by a treatment with pig liver esterase (PLE) which led to an enzymatic hydrolysis in the presence of potassium dihydrogen phosphate as the buffer and Arquad as surfactant. The release rate could be controlled by modifying the steric demand of the stoppers of the thread (dibenzyl or dibutylcarboxamido) which changed the dethreading release rate. Thus, the proper design would afford different desired rates of delivery of the scents, allowing it to accomplish distinct applications. An increment in the temperature also accelerates the rate of fragrance release. The inputs and outputs which control the release of the scents of the intertwined profragrances correspond to those of YES or AND molecular logic gates [138]. The enzymatic release of the scents at room temperature accomplished in the profragrances **11** bearing a butyl stopper can be consider as a YES single-input gate in which the input is the enzymatic hydrolysis and the output is the release of the target scent. Using another stimulus, such as increment of temperature, led to an AND gate, considering both enzymatic hydrolysis and heating as inputs and the fragrance release as output. This working mechanism is obtained in the case of the interlocked profragrances having a dibenzyl stopper, since both stimuli are required in order to release the fragrance. The operation of these interlocked profragrances paved the way to prepare a novel category of smart scent delivery systems based on molecular machines.

## 9. General Discussion

This section aims to compare between the different highlighted scaffolds which can be used for the controlled release of scents in a general manner (Table 1).

Polymers are by far the most implemented scaffolds for the controlled release of scents [27,28,29,30,31,32,33,34,35,41,47] with several available stimuli and vast structural possibilities (Table 1, entry 1). Natural polymers offer the advantage of biocompatibility and good affinity with fragrance of different chemical nature, both by physical and chemical interactions. However, if precise control over the release rate is intended, synthetic polymers play a key role. When such systems are employed, both polymeric profragrances and byproducts after releasing the target scent must be nontoxic. Although this type of scaffold is employed in some commercially available applications in the perfume and related industry [139,140,141], there are some issues to overcome when a precise control of the release rate is necessary for the desired application. At the laboratory level there are some systems which allow such control, but for an industrial application it is necessary to develop an effective scaling process and at a low cost.

While ionic liquids are not highly used in the development of profragrances, they offer the possibility of a dual functionalization [58], both in the anionic and cationic part (Table 1 entry 2). This could lead to the release of more than one fragrance for the development of applications in fine perfumery. Further development is still needed before carrying out industrial applications with these promising scaffolds.

The use of MOFs as controlled release systems of scents leads to long release times (Table 1, entry 3), which turns out to be very useful when applications in which a prolonged odor perception are required, such as air fresheners. However, most of the employed systems are based on sustained release [82,84,87,89,90], with the MOF acting as encapsulant which retards the aroma evaporation, but without a precise control over the process. The extensive use of stimuli-responsive organic struts to construct these metal-organic materials [142] leads to envision a future development of advanced implementations with a fine-tunable release operation.

Other systems, such as organic salts and coumarins (Table 1, entries 4 and 5) offer a fine-tuning of release rate and releasing operation modes which operate through daily life inputs in an easy manner [91,96,102,103].

Cyclodextrins also are suitable systems to accomplish a sustained release (Table 1, entry 6), highlighting their biocompatibility which had allowed several applications in the development of functional foods [111]. However, regarding controlled release of scents, the implemented systems led to a sustained release showing different release rates in function of the strength of the host–guest interactions of the target scent with these macrocycles [112,120]. Thus, a precise modulation of the release rate requires the incorporation of stimuli-responsive scaffolds to allow precise control over the release process by an external input.

Only one reported work on the use of rotaxanes for the controlled release of scents is available in the literature [137]. However, these systems pave the way to a precise control over scent release by a rational design of the different intertwined counterparts, leading to molecular machine-like operation modes. However, a lot of development needs to be carried out before a practical implementation of this proof of concept can be found. The major drawback for industrial applications of these systems is the yield over the formation of these interlocked profragrances (8–14%), so alternative templates should be explored in order to facilitate the rotaxane formation reaction.

In addition to the properties mentioned above, the selection of the release systems can be determined using release times as criteria. Thus, Table 2 includes the range of release times of scents which can be provided by the different type of scaffolds.

## 10. Summary and Outlook

This review article aims to highlight different available options in order to develop controlled release systems of scents, but it is not intended to offer a catalog in which all the reported examples are listed. Thus, different carefully selected recent examples provide an overview of the suitable options which can be used by researchers in the area.

One of the main research directions in the fragrance industry seeks to reduce the volatility of scents through the development of molecular systems which allow the slow release of these volatile compounds in a controlled manner. In order to develop applications which can benefit the consumer for everyday use, there is a series of requirements which must be fulfilled: (i) the fragrance precursors must be biocompatible; (ii) once the release of the scent occurs, the rest of the components which are generated must be nontoxic; (iii) the stimuli that cause the release of the scents must occur spontaneously in the context of the application conditions, without the need to use additional artificial sources, being desirably an effective operation through sunlight, hydrolysis by enzymes present in the human skin or the ambient humidity itself. Additional stimuli such as temperature increase can be crucial to achieve the desired efficiency for industrial or laundry applications, among other implementations.

The release time depends on the desired application, requiring an accurate rational design of the systems. However, due to the employed stimuli, a precise and reproducible release rate turns out to be difficult to achieve, since the intensity of the light or the percentage of water in the air vary depending on the ambient conditions. For this reason, a lot of efforts must be devoted in order to develop scent nanodispensers having an extremely precise control of the release rate. Thus, the possibility of using a wide range of molecular scaffolds greatly benefits the advancement of this area of research, which leads to forecasting a promising future in the development of enhanced functional delivery systems.

Fragrance and flavor release systems based on polymeric architecture have been the most widely employed, leading to applications in perfumery products, in textile manufacturing, or in the food industry. The interest in the preparation of new types of polymers allows to foresee that the progress in this subfield of materials science will lead to more sophisticated scent delivery systems in the future. The characteristics of polymeric scaffolds which have led to their predominance in the research field are the easy formation of micro- and nanocapsules, the wide range of polymers showing biocompatibility and the numerous functionalization possibilities.

Despite the predominance of the use of polymers as nanodispensers of fragrances, other molecular materials such as metal–organic frameworks have been postulated as highly tunable candidates for sustained release. The main advantage offered by MOFs is the vast design possibility which forecasts crystalline materials having almost limitless future prospects, resulting in tunable control of the pore size, which potentially facilitates the selective incorporation of specific aromas and the precise modulation over the release rate. If applications of MOFs are intended in the development of fine perfumes, functional foods, or body care products, among others, a nontoxicity of MOFs themselves, metal nodes, and distinct degradation products generated during the release process of the scent turns out to be a mandatory requirement.

In addition to the encapsulation methods using materials, the reversible formation of inclusion complexes using cyclodextrins has turned out to be a suitable approach to afford the stabilization of scents and their subsequent release. The main advantage of these macrocyclic molecules is their biocompatibility, which has led to interesting applications in food science. However, the association constants of the host fragrances with the CD must be high enough to stabilize the inclusion complexes, but also allow the adequate variations to accomplish the scent release under the application conditions. Thus, a careful selection of the CD derivative and the host scents is mandatory in order to achieve the desired implementation.

The covalent attachment of scent precursor fragments into profragrance backbones through the formation of cleavable bonds, such as ester bonds, is a highly effective strategy for scent release under realistic use conditions. Thus, ionic liquid-, pyridoxal acetal-, quaternary pyridinium surfactant-, and coumarin-based profragrances have been applied for the controlled release of scents using light or water inputs. This cleavable covalent bond approach paves the way to the use of a large number of small molecules for the development of stimuli-responsive scent release systems. Thus, an ester bond has been also introduced as a scissile element in rotaxane-based systems to accomplish release of scents by an AND or YES molecular logic gate-type operation.

The incorporation of mechanically interlocked systems as profragrances further expands the design possibilities since the functionalization of the different counterparts of the intertwined species is feasible. The employment of MIMs in the preparation of materials leads us to foresee future implementations of MOFs with intertwined ligands and interlocked polymers which will afford enhanced release properties. This research path will grow in parallel with the progress of artificial molecular machinery.

Although progress in the precise modulation of the rate of fragrance release is one of the milestones to overcome, the pool of molecular systems which can be used will undoubtedly lead to a great development, benefitting from the inherent interdisciplinarity of this research field, which merges materials science, supramolecular chemistry, synthetic chemistry, food chemistry, and scent chemistry, among other scientific areas. Thus, through this interdisciplinary approach the target release patterns with enhanced pleasant perception of some scents are expected by a full understanding of the physicochemical parameters of the release processes, and also the development of cargo delivery systems which operate in a very effective and selective manner.

## Figures and Tables

**Table 1:** General considerations of the different scaffolds employed for the controlled release of scents.

Entry	Type of Controlled Release System	Available Stimuli	Principal Property
1	Polymers	pH, light, humidity, temperature	Great structural range
2	Ionic liquids	pH, temperature	Potential development of dual systems
3	MOFs	Sustained release	Long release times
4	Organic salts	pH, humidity	Fine-tuning of release rate
5	Coumarins	Light	Sunlight-responsiveness
6	Cyclodextrins	Sustained release	Biocompatibility
7	Rotaxanes	Light, temperature, enzyme	Precise control over release

**Table 2:** Range of release times of scents provided by the different controlled release systems.

Entry	Type of Controlled Release System	Release Time Range
1	Polymers	Hours to months
2	Ionic liquids	Hours to days
3	MOFs	Hours to years
4	Organic salts	Hours to days
5	Coumarins	Hours to weeks
6	Cyclodextrins	Hours to months
7	Rotaxanes	Hours to days

## Data Availability

Not applicable.
